# Targeting the alternative oxidase (AOX) for human health and food security, a pharmaceutical and agrochemical target or a rescue mechanism?

**DOI:** 10.1042/BCJ20180192

**Published:** 2022-06-24

**Authors:** Marten Szibor, Christina Schenkl, Mario R. O. Barsottini, Luke Young, Anthony L. Moore

**Affiliations:** 1 Department of Cardiothoracic Surgery, and Center for Sepsis Control and Care (CSCC), Jena University Hospital, DE-07747 Jena, Germany; 2 Faculty of Medicine and Health Technology, Tampere University, FI-33520 Tampere, Finland; 3 Biochemistry & Biomedicine, School of Life Sciences, University of Sussex, Brighton BN1 9QG, U.K.; 4 Genomics and BioEnergy Laboratory, Institute of Biology, University of Campinas, Campinas – SP 13083-862, Brazil

**Keywords:** agricultural diseases, alternative oxidase, di-iron carboxylate proteins, mitochondrial diseases, pathogens, respiratory chain

## Abstract

Some of the most threatening human diseases are due to a blockage of the mitochondrial electron transport chain (ETC). In a variety of plants, fungi, and prokaryotes, there is a naturally evolved mechanism for such threats to viability, namely a bypassing of the blocked portion of the ETC by alternative enzymes of the respiratory chain. One such enzyme is the alternative oxidase (AOX). When AOX is expressed, it enables its host to survive life-threatening conditions or, as in parasites, to evade host defenses. In vertebrates, this mechanism has been lost during evolution. However, we and others have shown that transfer of AOX into the genome of the fruit fly and mouse results in a catalytically engaged AOX. This implies that not only is the AOX a promising target for combating human or agricultural pathogens but also a novel approach to elucidate disease mechanisms or, in several cases, potentially a therapeutic cure for human diseases. In this review, we highlight the varying functions of AOX in their natural hosts and upon xenotopic expression, and discuss the resulting need to develop species-specific AOX inhibitors.

## Introduction

**Figure 1.**
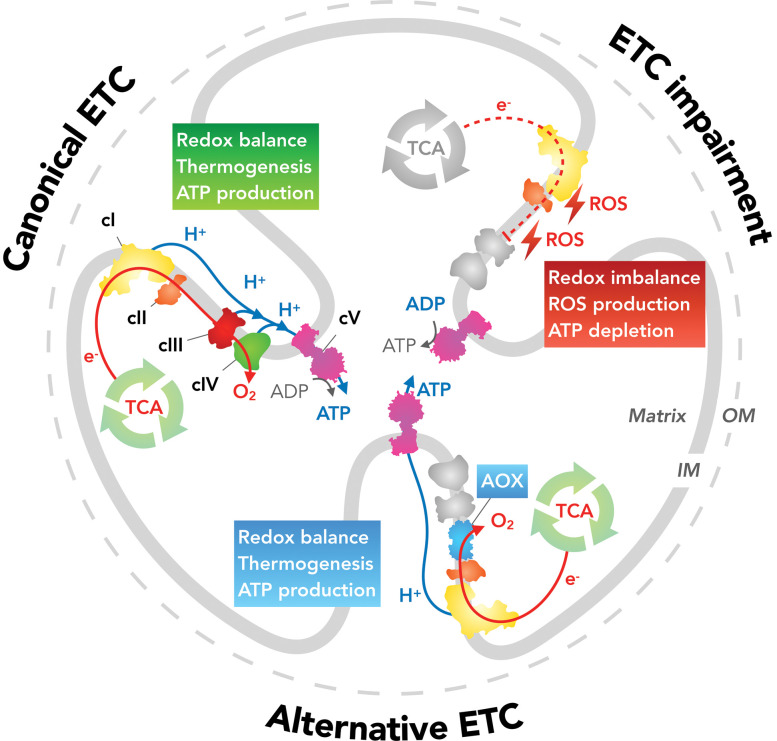
The canonical electron transport chain (ETC) consists of four oxidoreductase complexes namely cI (NADH:ubiquinone oxidoreductase), cII (succinate:ubiquinone oxidoreductase), cIII (ubiquinol:cytochrome *c* oxidoreductase or the cytochrome *bc_1_* complex) and cIV (cytochrome *c* oxidase). The transfer of electrons from respiratory substrates at cI and cII to oxygen at cIV is coupled to proton translocation across the inner mitochondrial membrane (IM) at cI, cIII, and cIV generating a H^+^ electrochemical gradient which is the main driving force (protonmotive force, *pmf*) for ATP production at cV (mitochondrial F_1_F_0_-ATPase). This coupled process is hence termed oxidative phosphorylation (OXPHOS). Oxidation of respiratory substrates, generated by the TCA cycle (tricarboxylic acid cycle or Krebs cycle), maintains the TCA and keeps upstream metabolic circuits operational. The main functions of the ETC include maintenance of cellular redox balance, heat production, carbon turnover for biosynthetic purposes and ATP generation by OXPHOS. If cIII and/or cIV are impaired, electron flux through the ETC is stalled, causing an arrest of the TCA cycle and upstream metabolic cycles, as well as proton translocation. This leads to redox imbalance, ROS production and eventually decreases OXPHOS. AOX (alternative oxidase) restores electron flux through the ETC and thus most mitochondrial and metabolic functions even though cIII and cV are bypassed.

Enzymes act as catalysts for chemical reactions and thereby enable living organisms to adopt to their environment and convert energy from nutrients ready for all vital processes such as growth, maintenance, or reproduction [1–3]. A class of enzymes that has gained particular importance in recent years are the alternative oxidases (AOXs). AOXs belong to a subfamily of di-iron carboxylate proteins which include methane mono-oxygenases, ribonucleotide reductases, fatty acyl desaturases, and rubrerythrin [4]. Although AOX is a long-established redox partner of the mitochondrial electron transport chain (ETC) (Figure 1), its exact function in the process of energy conversion has for a long time been debated. Initially, AOXs were thought to be restricted to plant mitochondria, with their main function being to maintain TCA cycle activity under conditions when energy is provided by photosynthesis [5]. Seminal work later discovered that in plants the protein is induced by different forms of stress, such as inhibition of electron flux through the ETC, excessive ROS production or, importantly, during pathogen attack [6,7]. With the advent of technologies allowing DNA analyses and the availability of genome sequences, as well as the availability of AOX-specific monoclonal antibodies, AOX has subsequently been shown to be present in most kingdoms of life [5,8–10] implying a prominent role of this protein in cell viability. Furthermore, evidence emerged suggesting that AOX evolved very early in evolution, probably from a common ancestor of prokaryotes [8]. Amino-acid sequence analyses revealed that the AOX protein is highly conserved, irrespective of the host organism in which it is expressed. The broad presence within various phyla and subphyla has been confirmed by a variety of techniques including bioinformatic identification of its sequence, gene expression data upon amplification by reverse-transcriptase polymerase chain reaction (RT/PCR) and/or functional assays. Indeed respirometry confirmed the presence of cyanide-resistant respiration [9], validated using relatively non-specific AOX inhibitors, i.e. salicylhydroxamic acid (SHAM) and propyl gallates (PG) [11,12]. It is clear that AOX is not only critical for respiratory activity in plants but also necessary for the survival of numerous phytopathogenic and parasitic organisms. Of particular significance was the finding that several protist organisms which are exceptionally pathogenic to humans contain an AOX such as trypanosomes [13], *Cryptosporidium parvum* [14], *Blastocystis hominis* [15] and the microsporidia [16]. The full extent of AOX distribution in prokaryotes is still relatively uncertain, but it is becoming increasingly clear that AOX is also widespread among proteobacteria, including *Novosphingobium aromaticivorans* [17], members of the *Vibrio* genus [8,18,19] and *Thiobacillus denitrificans* [8]. In addition to its wide distribution in plants and prokaryotes, AOX is present in phytopathogenic fungi such as *Blumeria graminis* [20], *Magnaporthe grisea* [20,21], *Septoria tritici* [22], *Ustilago maydis* [20,23], *Sclerotinia sclerotiorum* [24,25] and *Botrytis cinerea* [20,26] to name but a few. Other notable species expressing AOX include the invertebrate marine worms *Arenicola marina* [27] and *Sipunculus nudus* [28], the bivalve *Genkendia demissa* [9], the Pacific oyster *Crassostrea gigas* [9], and the sea squirt *Ciona intestinalis* [29,30]. Since vertebrates lost AOX from their genome during evolution, *Ciona intestinalis* is now the closest known relative to humans still expressing a functional AOX enzyme. Remarkably, AOXs in fungi and *Ciona* appear to facilitate similar stress responses as has been described for plants, namely the conversion of cellular energy, redox homeostasis, and the control of mitochondrial ROS content [27,30,31] (Figure 1). This role in stress response makes AOX a valuable therapeutic target in both agriculture and environmental protection and possibly also in in human infectious diseases. For this very reason, AOXs in general and the one isolated from *Ciona* in particular have been utilized as a means to correct ETC impairments in humans, and we suggest that their use *in vivo* may even represent a potential novel therapeutic treatment for mitochondrial diseases [29,32–36].

Here, we examine the various functions of AOX in selected host species and show how its expression may help to exacerbate the pathogenicity of the respective species. Furthermore, we discuss recent studies that use xenotopic expression of AOX to challenge the etiology of mitochondrial diseases in an attempt to provide a cure.

## Human pathogens that utilize AOX for pathogenicity

Numerous human pathogens express AOX, the activity of which facilitates a number of vital functions in the pathogen adaptation processes, which in turn can enhance their pathogenicity. One example is in *Trypanosoma brucei* (a causative agent of trypanosomiasis), where AOX plays a crucial role in adapting the metabolism to facilitate its life-cycle either in the tsetse fly (genus *Glossina*) or within the human bloodstream. Originally, it was thought that the bloodstream form of *Trypanosoma brucei* relied on AOX for ATP production by OXPHOS [37,38]. This seems counterintuitive considering that at this stage the pathogen is almost exclusively dependent on glycolysis for ATP production [39,40]. This notion is supported by the finding of a single mitochondrion with few flattened cristae in the bloodstream form [41] while a fully branched mitochondrial network is present in the procyclic insect form [39,40]. The switch in metabolism from oxidative phosphorylation to glycolysis and *vice versa* occurs rapidly and is likely regulated by substrate availability, eventually also leading to an altered mitochondrial proteome [42]. Whilst the procyclic form harbors all the ETC and Krebs cycle enzymes required for ATP production, the bloodstream form lacks the heme proteins required for respiratory complexes III and IV. In the bloodstream form of trypanosomes, two membrane-bound enzymes are expressed instead, i.e. glycerol-3-phosphate dehydrogenase and AOX, which together form a futile cycle rather than a fully functional ETC. Neither of these enzymes are protonmotive and hence cannot contribute to the protonmotive force (*pmf*), a prerequisite for mitochondrial ATP formation [39], suggesting that a contribution of AOX-mediated respiration to ATP production by OXPHOS in the bloodstream is an unlikely event. The actual role of AOX in the bloodstream form is rather to maintain redox balance, i.e. decreasing accumulating NADH levels thereby allowing glycolysis to continue to operate [40]. Interestingly, ATP inhibits trypanosomal AOX [40]. To avoid ATP-mediated inhibition of AOX and thus a stalling of the parasitic metabolism, the trypanosomal F_1_F_0_-ATPase hydrolyses ATP to pump protons across the inner mitochondrial membrane [40]. The paradoxic conclusion, therefore, is that the bloodstream form of *Trypanosoma brucei* indeed relies on AOX activity for ATP production, albeit via glycolysis rather than OXPHOS [37,38].

A further example of a pathogen in which a functional AOX has been discovered is the apicomplexan *Cryptosporidium parvum* [43]. *C. parvum* causes severe diarrheal disease (cryptosporidiosis), contributing significantly to mortality in children and immunocompromised individuals [44]. Unlike trypanosomes, *C. parvum* has a single-host life cycle in the intestine during which obligate asexual and sexual processes occur [45]. To date, no appropriate treatment exists. However, inhibition of AOX by SHAM and 8-hydroxyquinoline has been shown to inhibit the growth of this parasite *in vitro* [43] suggesting a vital role of AOX for its pathogenicity. The exact role of AOX in the pathogenicity is still unclear but as demonstrated for trypanosomes, its metabolism is largely glycolytic [46]. Interestingly, cryptosporidia have both aerobic and anaerobic metabolic pathways, which undoubtedly increases metabolic flexibility necessary for it to exist in a changing environment [44,47]. Notably, *C. parvum* possesses a degenerate mitochondrion and an incomplete F_1_-ATPase (only the α- and β-subunits are expressed [48]). It is thus tempting to speculate that these F_1_-ATPase subunits are expressed to control ATP content and consequently AOX activity thereby increasing both viability and pathogenicity of *C. parvum*.

A seemingly different situation is observed in *Blastocystis hominis*, a parasite generally considered to be an obligate anaerobe [15,49]. Intriguingly, it was discovered that *B. hominis* possess only mitochondria-like organelles with an incomplete ETC and a partial Krebs cycle alongside other metabolic pathways but does contain a functional AOX [49]. Since AOX uses oxygen as a final electron acceptor, its presence implies that oxygen fluctuations occur which represent a threat to the parasite. Indeed a recent study suggests that the blastocyst AOX can buffer oxygen fluctuations in the gut [15]. According to this study, AOX accepts electrons from ETC complex II (succinate:ubiquinone oxidoreductase/fumarate reductase) [15] and since neither complex II nor AOX are protonmotive, they form a futile cycle. Again, AOX cannot primarily contribute to ATP production by OXPHOS. A possible advantageous effect of expressing AOX could instead be that it prevents excessive mitochondrial ROS production, preserves the redox balance and/or influences the direction of important metabolic transport systems, such as the malate-aspartate shuttle (Figure 1). Eventually one or a combination of the proposed mechanisms may increase the pathogen's metabolic flexibility and thus the observed resilience of blastocysts.

AOX is also expressed in *Acanthamoeba* [50] which can cause a rare granulomatous amebic encephalitis involving the central nervous system that characteristically occurs in immunocompromised patients and nearly always causes death, or a painful keratitis resulting in blindness [51,52]. *Acanthamoeba* co-expresses two fully functional and competing quinol-oxidizing pathways, the phosphorylating canonical ETC and the cyanide-resistant AOX pathway [53,54]. Both pathways not only share the substrate (ubiquinol), but also interact kinetically, thereby directly or indirectly affecting the activity of the other. For instance, it was demonstrated that a direct protein–protein interaction exists between AOX and subunits of complex III [55]. Moreover, it has been shown that binding of GMP has a positive allosteric effect whilst the binding of ATP has a negative allosteric effect, and that both operate competitively, in that they overcome each other's effect [56,57]. Although the role of AOX in pathogenicity has not been fully elucidated, data suggests that AOX is induced in response to stress, e.g. during the encystment process [50], to buffer mitochondrial ROS generation [58] or to generate the heat needed to allow growth at low temperatures [59].

Other emerging human pathogens that broadly express AOX include the microsporidia, a group of obligate intracellular parasitic eukaryotes [16]. The identification of AOX in these parasites is particularly surprising because microsporidia were for a long time considered to be amitochondriate until remnants of mitochondria, now called mitosomes, were discovered [60]. A hallmark of microsporidia is that they have adapted perfectly to life as intracellular parasite reflected by a drastically reduced biological complexity such as loss of cell organelles and a massively shrunk genome [61–65]. Mitosomes lack a genome and genes encoding subunits of the ETC, the mitochondrial ATPase, and Krebs cycle enzymes appear not to exist [16,61]. Therefore, most microsporidia rely on glycolysis for ATP production, or are exclusively dependent on ATP import from the host as they even lack a fully functional glycolytic pathway [66]. Beside proteins required for the assembly of iron-sulfur clusters, mitosomes harbor a glycerol-3- phosphate dehydrogenase and, notably, an AOX [16,61]. The biological role of AOX in microsporidia likely depends on the stage in their respective life cycle [67], but it is reasonable to conclude that it plays a role similar to that in trypanosomes, namely to facilitate the recycling of reducing equivalents to keep glycolysis and/or other metabolic circuits operational. Whether an ATPase activity also exists to regulate AOX activity has yet to be determined.

AOX is also expressed in opportunistic human fungal pathogens, i.e. pathogens that do not represent a health threat unless an individual is immunocompromised or susceptible through other conditions, such as *Candida albicans* (candidiasis) [68,69], *Cryptococcus neoformans* (cryptococcosis) [70] and *Aspergillus fumigatus* (aspergillosis) [71]. The biological role of AOX in *Candida albicans* is of particular interest since its expression appears to be a reaction to different forms of increased stress resistance. In other words, the protein is expressed to protect against mitochondrial ROS generation during copper starvation [72], to decrease the susceptibility of the cell to antifungal drugs [73], or to prevent the loss of viability and cell wall rearrangements that eventually lead to increased recognition and uptake by macrophages [74]. A worldwide concern has recently emerged due to the discovery of *Candida auris* [75–77], a multidrug-resistant form of *Candida,* first isolated from a patient's auricle in Japan in 2009 [78]. This multidrug-resistant and primarily nosocomial pathogen has been associated with fungemia, ventriculitis, osteomyelitis, malignant otitis and otomastoiditis, and complicated forms of intra-abdominal infections, pericarditis, pleural effusions, and vulvovaginitis in a broad range of patients, including immunocompromised individuals [75–77]. Since its first description, *C. auris* infections have been reported in India, South Africa, Kuwait, Venezuela, Brazil, the United States, Colombia, Pakistan, Spain, Germany, Israel, Norway, Oman, and the United Kingdom [75–77]. The latter is the first description of a hospital-acquired transmission, which has led to a greater outbreak in a European country, adding to the notion that this multidrug resistant pathogen is capable of transmission in the health care setting potentially causing serious infections of a global concern. Particularly alarming is the finding that *C. auris* appears not only resistant to fluconazole [79], a drug commonly used to treat candidemia, but also to the other three main classes of antifungal drugs, azoles, echinocandins and polyenes [76].

Whilst metabolic flexibility is considered a key characteristic of *Candida* spp. enabling it to swiftly adapt to varying enviromental conditions and host niches [80], little is known about specific virulence factors of *C. auris*. Since AOX enables a metabolic stress resistance of *C. auris*, we have previously suggested that AOX inhibitors may become a safe and effective treatment approach and thereby could become a ‘game changer' in the clinic [69]. In support of this notion, functional studies with chemical or genetic silencing of AOX activity have provided further insights into the role of this enzyme in the virulence and pathogenicity of numerous human pathogens while bypassing the mode of action of conventional drugs. For instance, *Paracoccidioides brasiliensis* is one of the causative agents of paracoccidioidomycosis, and treatment of *P. brasiliensis* [81] with the non-specific AOX inhibitor SHAM prevented the transition from the mycelial to the yeast form, a critical step for host colonization. Likewise deletion of the *C. neoformans* [70] *aox1* gene, which is induced at 37 °C presumably after sensing the human body temperature, led to a decrease in *C. neoformans* virulence in a murine model. Finally, silencing of the *A. fumigatus* AOX has led to a decreased viability in the presence of ROS and a higher rate of killing by macrophages [82].

As an increasing number of mechanisms of action of antibiotics and antifungal drugs against human pathogens are bypassed, the presence of multidrug-resistant organisms thus poses a serious threat to human welfare. AOX is emerging as a suitable pharmacological target. To date many compounds have been reported as inhibitors of the AOX, with the earliest and most used historically being the alkyl-gallate and hydroxamic acid derivatives from the 1980s (Table 1). Unfortunately, the low potency and low selectivity of most compounds have prevented their clinical use [83], which is now changing in large part due to advances in the biochemical and structural characterization of purified AOXs. The development of potent AOX inhibitors which are specific and exhibit broad-spectrum species sensitivity, act in the nM range, and have no or minimal side effects appears to be a viable approach. A number of such promising new molecules have been identified and tested both in our and other laboratories and are currently awaiting further clinical assessment. Latest advances and differences in the mode of action of individual inhibitors have recently been reviewed in detail elsewhere and will not be part of this review [84].

**Table 1:** AOX inhibitors or inhibitor classes

Compound	PubChem CID	Structure	References
Benzoic acid derivatives	135	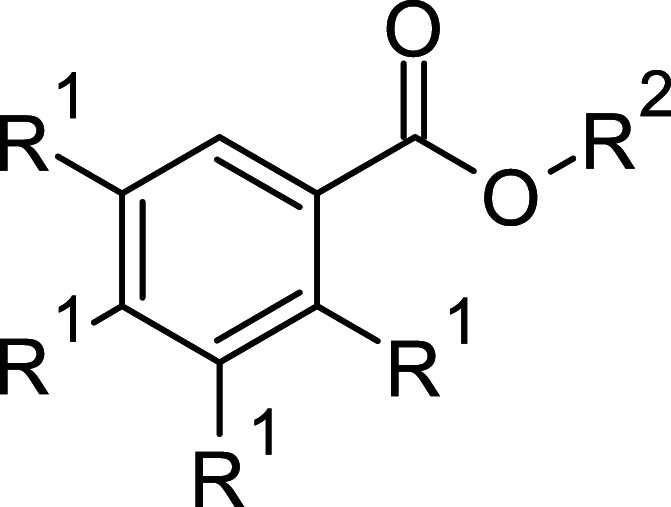	[123,234–236]
Ascofuranone derivatives	6434242	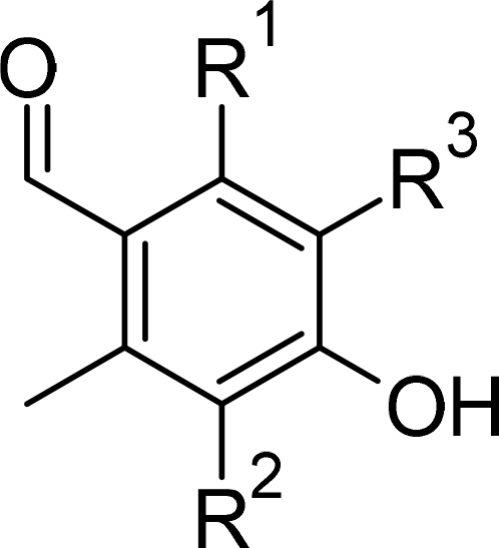	[69,122,125,127,237]
Decyl-Aurachin C	6439171	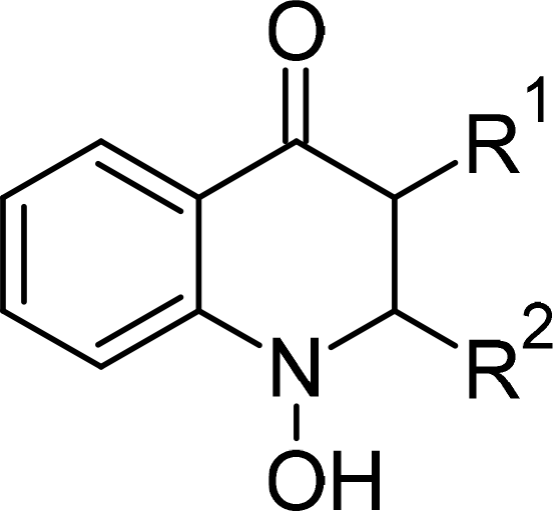	[238]
Chloroquine	2719	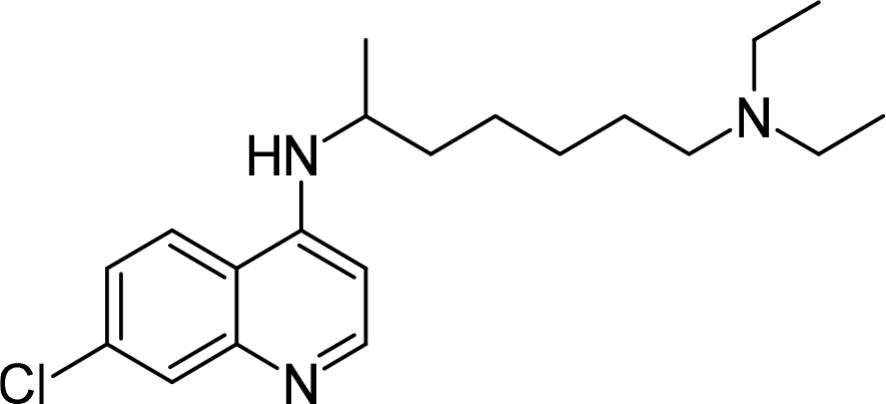	[239]
Coumarin derivatives	5650046	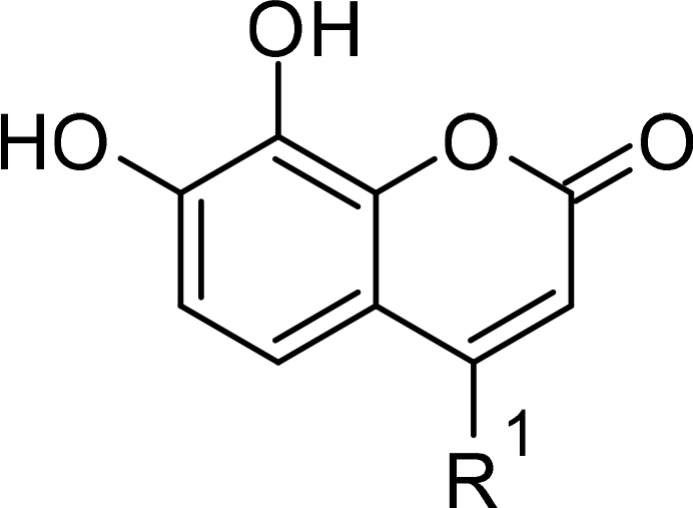	[240]
Disulfiram	3117	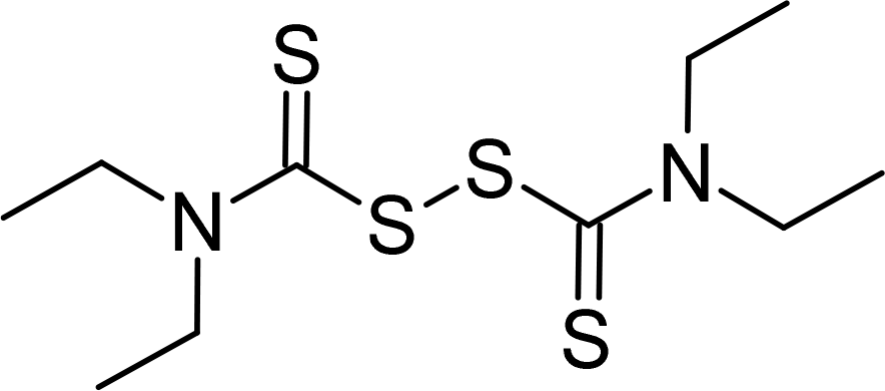	[241]
Ferulenol	54679300	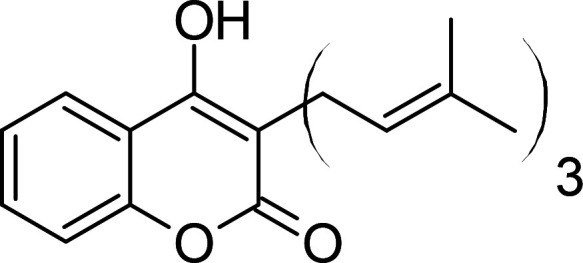	[126]
Gallic acid derivatives	61253	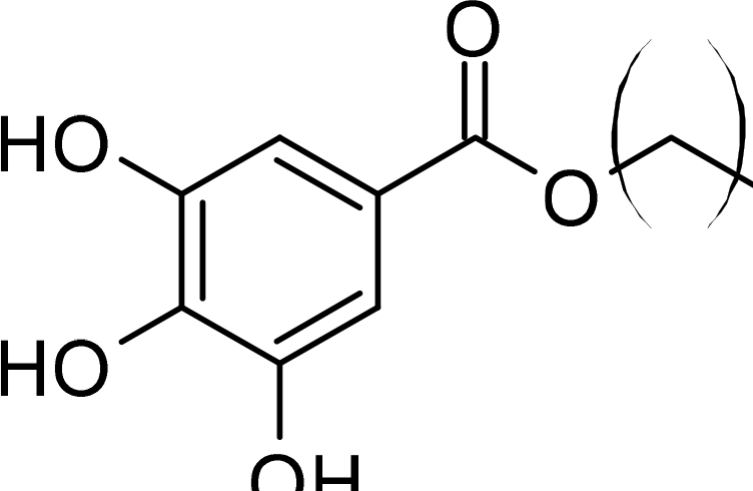	[11,235,238,242]
Hydroxamic acid derivatives	66644	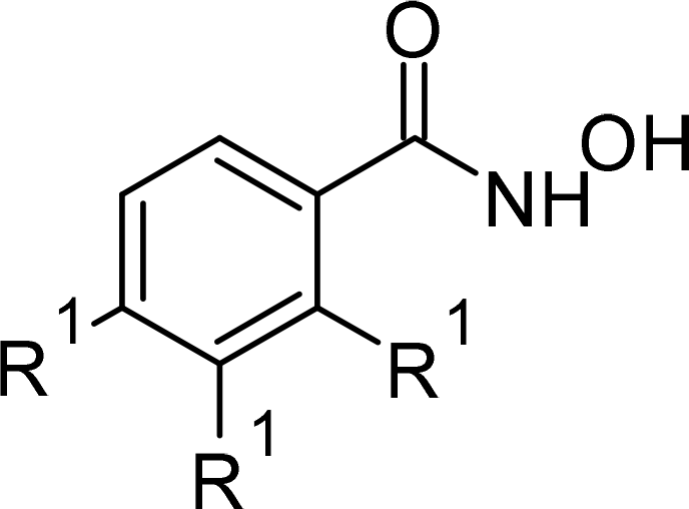	[235,242,243]
Linoleic acid	5280450	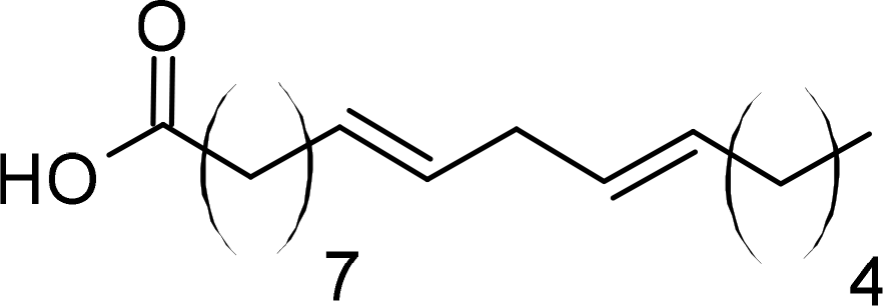	[244–246]
N-phenylbenzamide derivatives	3532749	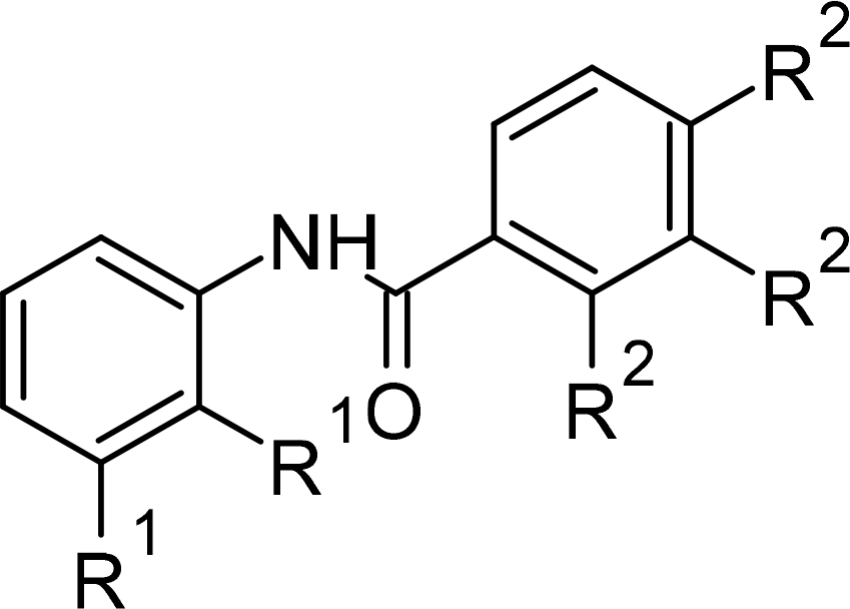	[98,247]
5-n-undecyl-6-hydroxy-4,7-dioxobenzothiazole	3016416	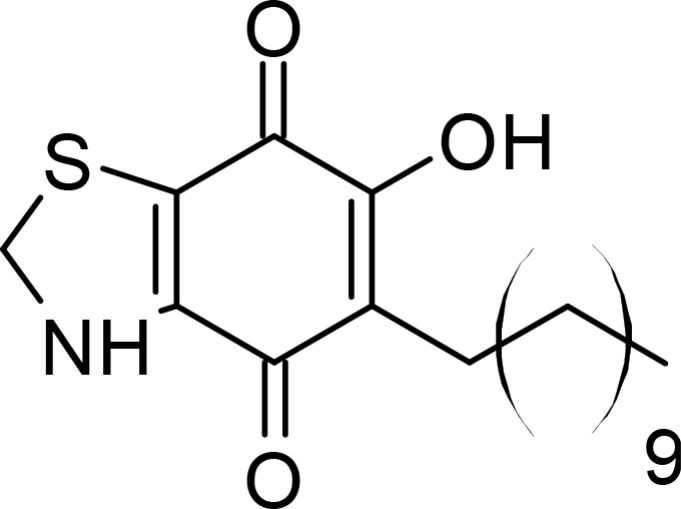	[244,248]

Structures column depicts core inhibitor structures, with R groups indicating positions of functional group manipulation used within the reference list.

## Possible benefits of AOX inhibitors in agrochemicals

The rising presence of fungal infections is also recognized as a global threat to food safety in agriculture, which endangers both ecosystem and human health [85]. Fungal infections which have a negative impact on food quality are well-described for cereal crops. For instance, *Fusarium graminearum* in wheat [86], *Magnaporthe oryzae* in rice [87], *Ustilago maydis* in maize [88], and *Sclerotinia sclerotiorum* in potatoes [89] and soybean [90]. Such infections are also devastating non-cereal crops, such as *Moniliophthora perniciosa* in cocoa [91,92], *Mycosphaerella fijiensis* in banana [93], *Venturia* spp. in apple [94], and various others [95]. Disturbingly, a simultaneous occurrence of severe epidemics in all five cereals would most certainly have a catastrophic impact on food security, as there would not be enough food available for the world's growing population. Analagous to the aforementioned human pathogens, a great number of phytopathogenic fungi also use AOX to escape stress response mechanisms thereby enhancing pathogenicity [21,25,96,97].

Unfortunately, only very few studies have addressed the biochemical differences between the various AOX enzymes and the different roles that fungal AOXs may play in plant pathogenesis. Hitherto, it has been demonstrated that in the cocoa pathogen *M. perniciosa*, AOX is necessary for spore germination and host infection [98] while *M. grisea* virulence in barley leaves appears to use different mechanisms [21]. A more indisputable role, and perhaps more relevant from an agronomic perspective, is attributed to the importance of AOX as a source of resistance to fungicides used as crop protection agents. The best-studied case is the inhibition of complex III (cytochrome *bc1* complex) of the ETC by strobilurins and other so-called quinone-outside inhibitor (QoI) fungicides [99]. Due to the functional bypass of the cytochrome *bc1* complex, AOX can restore mitochondrial functions (Figure 1) and sustain fungal growth. Unsurprisingly it has been demonstrated that the inhibition of both pathways, the canonical ETC and the alternative pathway, is a promising approach to control several phytopathogens of agronomical importance. For instance, both *Septoria tritici*, a fungus that causes major leaf spot diseases in wheat [96], and *Gaeumannomyces graminis* var. *tritici* [100], the wheat ‘take-all' fungus, express AOX specifically when treated with respiratory inhibitors, thereby facilitating a fungicide strobilurin-resistant respiratory pathway. Similar results have been observed with *Magnaporthe grisea* [101], *Sclerotinia sclerotiorum* [25], *M. perniciosa* [97,98], *Venturia inaequalis* [102,103] and *Microdochium majus* [104]. QoI fungicides have been extensively used in the agriculture since the 1990's especially following the discovery of strobilurin (a natural compound isolated from the fungus *Strobilurus tenacellus*) and the development of synthetic analogs with enhanced field performance [99]. Azoxystrobin became the top-selling fungicide as little as four years after its launching and within 10 years strobilurins represented 20% of worldwide sales [105]. An alarming recent observation, however, is the increasing identification of field isolates resistant to strobilurins which at least in part can be attributed to AOX expression. Moreover, it has been shown in *M. grisea* that AOX allows fungal survival long enough for the emergence of point-mutations in the cytochrome *bc1* complex, such as the change of glycine 143 to alanine (G143A), thereby rendering azoxystrobin completely ineffective [106,107]. AOX has also been associated with resistance to fungicides using other modes of action such as procymidone, fluconazole and the imidazoles miconazole, econazole and ketoconazole [73,108]. Mechanistically this may be due to the contribution of AOX to metabolic and redox homeostasis under stress conditions, and/or ROS scavenging. As with QoI fungicides, it is likely that new resistant mutants will evolve after prolonged exposure to those fungicides. Since fungicides play a key role in the control of diseases in crops and are thus a vital part of ensuring global food supply and safety, the increasing resistance to fungicides not only threatens the efficacy of individual fungicides but is also a major cause of reductions in crop yield and subsequent environmental degradation.

A further aspect which deserves attention is the possible role of AOX in the production of harmful mycotoxins by food colonizing fungi which can occur in the field or during post-harvest storage. In addition to AOX allowing fungal growth as discussed above, which by itself can increase mycotoxin incidence, this enzyme may contribute to the metabolic pathways associated with mycotoxin production. There is a known positive correlation between AOX expression in *Acremonium chrysogenum* [109] and *Aspergillus nidulans* [110,111] and the production of cephalosporin C and sterigmatocystin, respectively. This implies that AOX may have a broad role in fungal secondary metabolites production, some of which can have important effects on the human welfare. For instance, sterigmatocystin is a precursor to a skin-permeable carcinogen aflatoxin B1 produced mainly by *Aspergillus flavus* but also by other *Aspergillus* species [112]. Since mycotoxins can cause serious health issues including cancer, immune system suppression, liver failure and congenital diseases, regulatory bodies around the world have created strict guidelines for mycotoxin contamination levels. In the European Union the maximum accepted level of aflatoxin B1 in several nuts and cereals is precautionarily restricted to 2 to 12 parts per billion (ppb), but it could be as low as 0.1 ppb in foods destined to infants (European Commission Regulation No 1881/2006 Dec 19, 2006; https://t1p.de/8t67). Health and economic losses, however, may not only occur through direct contact with human beings but also by contamination and killing of poultry and livestock. In addition, many mycotoxins are highly stable meaning that they may remain present even after the toxin-producing fungus has been eliminated during the food processing [113–115].

Because AOX is present in both the fungal pathogen and the infected plant, putative AOX inhibitors that can be approved as fungicides must have exceptional specificity to inhibit the pathogen without harming the host, and this specificity must be accompanied by high efficacy and (food) safety. A key challenge in understanding the structure-function relationship of AOX has been the identification of its substrate- and inhibitor-binding site and its mechanisms of catalytic action. A detailed knowledge of the nature of this binding site is thus important, since it will reveal if there is a common architecture for the binding of its substrate or an inhibitor, thereby providing unprecendented insights into differential mechanisms of binding. Only such knowledge will allow the rational design of phytopathogenic and anti-parasitic drugs that target AOX in a species-specific manner.

## Structural considerations

**Figure 2.**
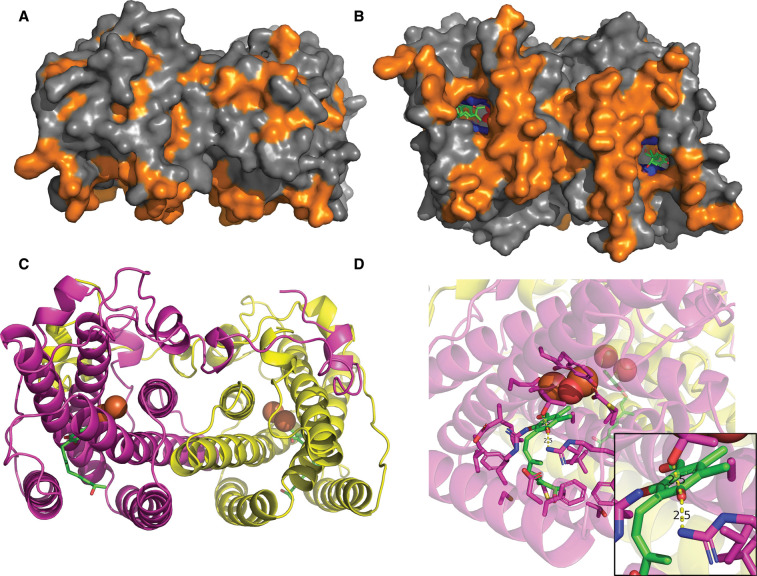
Crystal structure (PDB:5ZDR) of the alternative oxidase (AOX) dimer. (**A** and **B**) Surface model of the dimer showing hydrophobic residues in orange, hydrophilic in grey. (**B**) shows the entrance to the hydrophobic cavity in the membrane associating side of the protein, with the inhibitor depicted as green sticks and a leucine ‘gate', which controls inhibitor orientation, colored blue. (**C**) Helical structure of the dimer in the same orientation as **A**, with monomers colored magenta and yellow, respectively. Inhibitor can be seen as green sticks to show position of the hydrophobic cavity between helices 1 and 4 leading directly to the di-iron core, which shown as orange spheres. (**D**) The binding pocket within the protein, showing all amino acids within 5 Å of the inhibitor. Key hydrogen bonding between the phenol, Arg-118 and Thr-219 shown as yellow dotted line (inlet).

AOXs are self-folding di-iron carboxylate proteins harboring four highly conserved ExxH motifs, a characteristic of the common iron binding sequences found in non-heme iron binding proteins [116,117]. Initial AOX models proposed that a four-helix bundle acts as a scaffold to hold the iron core, the di-iron center being ligated by three glutamates, two histidines and one aspartate comparable to the binding sequences of methane monooxygenase or ribonucleotide reductase (Figure 2). These initial models also included two additional transmembrane helices with hydrophobic sections in the mitochondrial intermembrane space that were initially thought to function as substrate binding domains. Further modeling removed the transmembrane region in favor of a simpler association with the membrane based on hydrophobicity plots, and the proposed iron binding aspartate residue was replaced by a glutamate to evenly space the di-iron core binding across all four helices of the bundle [118,119]. Yet, it took another 13 years for the first AOX crystal structure to become available, largely due to the problematic nature of purification of membrane bound proteins in a stable form. Initial crystallization attempts of the trypanosomal AOX (often referred to as TAO) using digitonin were unsuccessful [120], but later improvements using n-octyl-β-d-glucopyranoside [121] increased both activity and stability of the protein, allowing for generation of crystal structures to a resolution of 2.3 Å [122]. Notably, the current structure lacks a section of the N-terminal region due to its ‘unstructured' nature which is thought to be the mitochondrial targeting sequence as its removal does not affect functionality of the recombinant protein [123]. Although attempts to apply these techniques to AOXs from other species [124,125] have been successful in generating stable purified protein, they nevertheless have failed to generate refracting crystals of sufficient quality for crystallography. Despite this shortcoming, most structural predictions previously made were proven to be correct, with the overall structure of TAO being that of a di-iron protein consisting of six long α-helices and four shorter α-helices, the metal core being ligated by four glutamates residues, E123, E162, E213 and E266, and two histidine residues, H165 and H269. A striking feature of the protein is its apparent crystallization in a dimeric form, with each of the monomers aligning in an antiparallel fashion. Given the conserved nature of the dimer interface across all AOX species and the symmetrical nature of the inter-monomer interaction of the unstructured N-terminal arm this arrangement is unlikely to be an artifact of the crystallization procedure, with the protein behaving as a structural dimer.

One of the most important structural features for the protein with respect to both its species-specific functionality and inhibition is the hydrophobic substrate cavity. This cavity is located between the α1 and α4 helices and leads directly to the di-iron core from the proposed membrane binding region [122] providing quinol, the substrate for AOX, direct access from the lipid bilayer to the reactive metal core. Whilst there is no crystal data with the substrate bound, numerous structures are available with inhibitors, such as colletochlorin B, AF2779OH or ferulenol, bound within the cavity [122,126], all of which have been shown to behave as transition state analogs indicating that the binding-site is highly likely to be the quinol binding site. Moreover, mutational analyses of the conserved residues lining the cavity has highlighted the significant changes in quinol kinetics that accompany slight changes to the amino acid composition, demonstrating the importance of this region to substrate/inhibitor affinity. Consequently, this region is of particular importance for the development of inhibitors for the control of pathogenic diseases mentioned above.

The trypanocidal activity of ascofuranone has been known since the mid 1990's, however, its low yielding chemical synthesis and low efficacy in blood clearance without the need for significant concentrations of glycerol led to a loss of scientific interest [127–129]. More recently there has been a significant resurgence in interest with this compound and its analogs, with numerous structure-function studies [130,131] and QSAR modeling work from the TAO crystal structure [132]. These studies highlighted the importance of the aldehyde functionality and the need for a hydrogen bond acceptor in the *para* position from the hydrophobic chain. In addition to ascofuranone-like compounds, the gallates are also considered to be effective inhibitors in high nanomolar region (Table 1). Recent developments in the gallate analogs (4-hydrobenzoates) show significant improvements in efficacy with the addition of TPP+, a charged lipophilic cation, aiding in targeting the compound to the mitochondria. An extensive review of all alternative oxidase inhibitors known to date, with a particular focus on their efficacy against TAO, however, can be found elsewhere [84].

Although, there is crystal data for the trypanosomal variant, significant homology modeling has been performed on AOXs from fungal [125] and human pathogens [15,133]. Of note, in most of these variants studied to date, the predicted cavity size appears decreased in comparison with TAO. Such a decrease in cavity size would significantly decrease the ability of compounds to inhibit the enzyme. This is especially apparent with ascochlorin, which, while active at nanomolar concentrations in TAO has its efficacy reduced 1000-fold when tested against the AOX from *M. perniciosa* [125]. Thus, effective inhibition of TAO has been widely studied, but that such inhibitors are also effective against fungal targets is still under investigation.

## Gene transfer for therapy

In addition to the role of AOX in enabling human and fungal pathogens to seek opportunistic ways of causing diseases, there are also perspectives for the use of AOX to challenge disease paradigms of human pathologies. One such emerging opportunity is within the area of AOX gene therapy, in attempts to overcome mitochondrial dysfunctions [134]. However, besides the potential therapeutic benefits of xenotopic AOX expression [35,36], one must be aware of possible side effects and pitfalls [29]. An obvious threat to viability could be that mitochondrial ATP production decreases due to the catalytic engagement of AOX and the consequent bypass of two of the three energy-conserving sites in the ETC (Figure 1). This could counteract putative beneficial effects such as the restoration of electron flux through the ETC, the redox balance, or heat production. Indeed, we found that xenotopic expression of AOX was deleterious in two independent mouse disease models, namely a model of mitochondrial myopathy based on skeletal muscle-specific ablation of the complex IV subunit COX15 [135] and in the postischemic heart [136]. In both, impaired signaling and maladaptive organ remodeling appeared to be the consequence of AOX expression. Yet, AOX is currently being considered for gene therapy in mitochondrial diseases ranging from Alzheimer's disease to emphysema and endotoxemia [137–139] after previous studies indicated that the symptoms of a disease may be alleviated or the condition can be completely cured. Notably, AOX gene therapy does not necessarily restore ATP levels [135]. Instead, it may reactivate respiratory complex I, thereby indirectly re-establishing the mitochondrial membrane potential which itself is indispensable for ATP production [140]. To date, AOX has been shown to efficiently bypass chemical inhibition of the ETC in flies [141] and mice [142,143], to rescue from lethality in cytochrome *c* oxidase (COX) deficient flies [141,144], and to partially rescue locomotor degeneration in flies exhibiting Parkinson's-like symptoms [141,145]. More recently it was shown that AOX can mitigate beta-amyloid production [137] (a causative agent of Alzheimer's disease) and toxicity in the fly and human cultured cells, and AOX also decreased lethality in an endotoxemia model of the mouse [138] and alleviated detrimental lung remodeling upon toxic smoke exposure [139].

## AOX to study Parkinson's disease in flies

Parkinson's disease (PD) is a neurodegenerative disease with an adult-onset that is characterized by the degeneration of dopaminergic neurons in the *pars compacta* of the substantia nigra. The dopaminergic neurons communicate with neurons of the basal ganglia by dopamine as a neurotransmitter and collectively are responsible for the fine tuning of movements. Consequently, clinical hallmarks of PD are slowness of movement (bradykinesia), rigidity, tremor, and the inability to maintain an upright position (postural instability). A mitochondrial involvement in the pathology has been postulated, since pharmacological inhibition of OXPHOS complexes such as the respiratory complex I inhibitor and neurotoxin l-methyl-4-phenyl-l,2,3,6-tetrahydropyridine (MPTP) [146–148] and the potent respiratory complex IV inhibitors carbon monoxide (CO) and cyanide produce symptoms that mimic PD to a great extent [149,150]. Furthermore, deletions in mitochondrial DNA have been found in the striatum of PD patients, as well as defects in OXPHOS due to mutations in nuclear encoded subunits of respiratory complexes I and IV in isolated mitochondria from muscle biopsies of PD patients [151] supporting the assumption of a mitochondrial involvement. Together, these findings have nurtured the idea of PD being a mitochondrial disorder.

To ultimately identify the position of mitochondrial OXPHOS dysfunction in the hierarchy of PD, the combination of animal models and genetic tools became inevitable. One such approach used the fly model, *Drosophila melanogaster*, in which the catalytic subunit of the mitochondrial DNA polymerase was down-regulated by RNAi technology [145]. Expectedly, this down-regulation disturbed ETC function. The use of cell-specific drivers allowed an ablation in specific neurons, e.g. dopaminergic neurons. This resulted in adult onset of progressive dopaminergic neurodegeneration and premature ageing with age-related motor deficits. Interestingly, the xenotopic expression of AOX, and partial restoration of PINK1/parkin signaling or Drp1, rescued the observed phenotype [145]. AOX restored ATP levels which argues for an increased electron flux through complex I, and more importantly, preventing dopaminergic neurodegeneration. This is a strong direct indication for a causal involvement of OXPHOS dysfunction in PD and subsequently a novel treatment option directly on-site of dysfunction for a disease that so far escaped a proper treatment approach.

Further evidence for a positive effect of AOX on the course of PD arose in another *Drosophila* disease model, the *dj-1b* mutant [141]. DJ1 is a highly conserved protein that was previously identified as a disease gene causing autosomal recessive early-onset parkinsonism [152]. The exact mechanism of DJ1 action in the pathology is not fully understood, but DJ1 was thought to affect ROS signaling thereby inducing apoptosis. Interestingly, the exceptional susceptibility of dopaminergic neurons to mitochondrial ROS contrasts to a relatively low mitochondria mass [153], although this may actually reinforce an OXPHOS dependence. In support of this, loss of respiratory complexes I and IV was detected in cultured neurons and forebrain samples from DJ1 knockout mice, suggesting energetically inefficient mitochondria and increased ROS production as a likely cause [154]. Another recent observation provided an additional perspective. It was found in brain mitochondria from DJ1 knock-out mice that respiration-dependent hydrogen peroxide consumption was increased [155], rather than decreased, which was attributed to increased activity of mitochondrial thioredoxin and glutaredoxin, and higher total GSH levels. A possible explanation for the seemingly counterintuitive finding may come from a study using an alternative genetic model, *Dictyostelium discoideum*. The results showed that DJ1 controls extramitochondrial functions such as phagocytosis and, to a lesser extent, pinocytosis under physiologic conditions [156]. Loss of function may thus only indirectly affect mitochondrial respiration which, nevertheless, may phenocopy conditions of OXPHOS deficiency. Of course, it is equally possible that different forms of PD exist based on entirely independent etiologies that, nevertheless, result in very similar disease states again exemplifying the complexity of PD. Irrespective of the precise underlying mechanism, AOX rescued *dj-1b* fly mutant [141], which is consistent with the idea of a disrupted ETC and subsequent up-regulation of mitochondrial ROS levels.

## AOX to study Alzheimer's disease in flies

Alzheimer's disease (AD) is the most common form of dementia based on a chronic neurodegenerative process that worsens over time [157,158]. A clinical hallmark of AD is the difficulty to remember recent events whilst older information remains temporarily accessible. Over time AD patients virtually lose their personality. Since vital body functions also decline the mean survival time from the onset of dementia ranges only from 3.3 to 11.7 years [159]. The etiology of AD is only incompletely understood. It is known that the risk of developing AD rises with age and thus the lifestyle may be an etiologic factor. Although, due to the long incubation time, it is difficult to identify cause-and-effect relations it is assumed that cardiovascular fitness is a protective factor against a cognitive decline [160,161]. Besides life-style factors, there is a documented genetic risk for late-onset and early-onset AD in carriers of apolipoprotein E4 (APOE4) [162]. While APOE is synthesized upon neuronal damage or stress and thus represents a regular repair mechanism, its conformation causes the generation of adverse neurotoxic fragments via proteolysis [163]. APOE4-derived fragments have a negative effect on the cytoskeleton and, of note, also cause mitochondrial dysfunctions. Furthermore it has been suggested that APOE is involved in clearance of amyloid beta (Aβ) peptide from the brain and that different APOE alleles may contribute differentially to the clearance [164]. Interestingly, while it is widely assumed that toxicity arises mainly from the extracellular insoluble protease-resistant fibrils, this concept has been disputed [165]. It appears that oligomeric Aβ peptides can disrupt the integrity of cell membranes [166] and, indeed, injected amyloid peptides impair synaptic transmission and induce cell death and cognitive decline if able to aggregate [167]. This would make the process of aggregation and subsequent membrane interaction the toxic event and not the passive presence of fibrils [168]. Toxicity may thus be primarily due to detergent-like properties on membranes and disrupted ionic homeostasis [169,170]. This mechanism may equally underlie the observed sensitivity of mitochondria to amyloid peptides [171]. Indeed, Aβ peptides impair cellular autophagy including mitophagy [172]. Interestingly, Aβ peptides are imported into mitochondria using the classical import machinery [173,174], which then has a number of effects [175]. For instance, an impairment of respiratory complex IV (cytochrome *c* oxidase) [171,176] has been demonstrated to be due to direct interaction in association with Aβ accumulation [177,178] which supports *postmortem* observations in brains of AD patients [179,180]. The proposed Aβ-induced disruption of electron transport through the ETC may also explain an observed decrease in the TCA cycle enzyme α-ketoglutarate [181] and an increase in ETC-related ROS production [182,183].

Despite the progress in understanding underlying disease mechanisms, no treatment has thus far has been developed which could stop or reverse the loss of cognitive functions. Based on current knowledge, however, mitochondria appear as a valuable target to shift fate [184,185]. This was recently tested in different models of AD pathology [137]. Specifically, AOX acted as antioxidant and thus decreased Aβ40 production which was induced in human HEK293-derived cells by the cytochrome *bc1* complex inhibitor antimycin A. Furthermore, AOX partially alleviated a neurodegenerative phenotype, i.e. a decreased lifespan in fly models expressing human Aβ peptides specifically in neurons. This improvement in survival was associated with a decrease in marker expression indicative of oxidative stress pointing towards disruption of electron flow through the ETC and thus induced ROS production as causative factor for the development of AD.

## AOX to decrease lethality due to endotoxemia in the mouse

Sepsis is defined as ‘life-threatening organ dysfunction caused by a dysregulated host response to infection' [186]. Sepsis-related organ failure has a high prevalence of morbidity and mortality, particularly associated with age, frailty, and severe comorbidities [187,188]. In the United States, for example, sepsis affects ∼1.7 million adults each year, and is thought to account for more than 250 000 deaths [187,189]. It has been estimated that sepsis occurs in up to 50% of hospital admissions eventually leading to death [187,189,190]. Unfortunately, sepsis survivors have also an increased risk of developing cognitive impairment and cardiovascular disease resulting in a shorter life expectancy [191]. Despite decades of research there is still no causal treatment option for sepsis [192]. Part of the reason for the failure of drug development is that molecular mechanisms underlying the development of sepsis are still ill-defined likely due to the complexity and systemic nature of the condition. One concept proposes that a so-called cytokine storm systemically dysregulates several signaling cascades thereby affecting all organs simultaneously [193].

The complex network of dysregulated signaling cascades observed during sepsis affects all levels of cellular metabolism and hence organs which rely heavily on OXPHOS, such as the heart, are particularly sensitive [194]. Recently, evidence emerged that mitochondria act not as helpless bystanders of a greater disturbance but are rather active players in the process. Due to conflicting results, the exact mode of impairment and the hierarchy of mitochondria in the pathology are still under debate [138,195–197], so-called mitochondrial interventions (often referred to as metabolic resuscitation) [198] have been identified as a potential therapeutic approach. Seemingly paradoxical, mitochondrial biogenesis is not necessarily able to recover mitochondrial respiration in sepsis [196]. In the liver, this may be explained by the stress-induced expression of nitric oxide (NO), carbon monoxide (CO) and hydrogen sulfide (H_2_S) [199], all three being potent inhibitors of complex IV [200–202]. Although therapeutically valuable at low or moderate levels [203,204], nitric oxide, carbon monoxide and hydrogen sulfide levels are in the context of pathologies significantly increased [202,205,206]. Given that hepatic parenchymal injury is critical for the development of the sepsis-related multi-organ failure, and respiratory disruption is the leading mitochondrial impairment in sepsis, it seems possible that increasing mitochondrial mass alone is not sufficient to overcome such potent inhibitors. Instead, mitochondrial biogenesis may have the opposite effect since more ROS may originate from a more dysfunctional ETC, possibly even accelerating multi-organ failure and septic shock. Conversely, protective effects have been described for antioxidants targeted specifically to the mitochondrial compartment, measured as a decrease in cytokine levels and oxidative stress while markers of mitochondrial and organ functions improve [207].

Cardiac contractile dysfunction is another important component towards fatality during sepsis. While systolic dysfunction paradoxically predicts a better outcome, diastolic dysfunction is a common and major predictor of mortality in sepsis [208]. Again, impaired OXPHOS was identified as the driving trigger of sepsis-associated myocardial depression and specifically myocardial complex IV has been shown to be impaired [209]. Supplementation with exogenous cytochrome *c* partially restored complex IV activity and improved cardiac function [210,211]. Interestingly, myocardial cell death is rare in sepsis and contractility is fully restored in survivors, arguing for a functional rather than structural impairment [212,213]. A phenomenon which is compatible with exposure to high levels of respiratory inhibitors as being the underlying mechanism.

Overcoming mitochondrial respiratory impairment has been identified as the key outcome of clinical and biochemical organ failure. Published data indicates that AOX expression provides protection against septic shock, based on mitochondrial protection or acceleration of the recovery process through stimulation of mitochondrial activity, e.g. in a mouse model of LPS-induced endotoxemia [138]. Mechanistically, it was demonstrated in isolated bone-marrow-derived macrophages that AOX prevents succinate-driven reverse electron transport (RET) and dramatically decreases mitochondrial ROS. Such results suggest that AOX may have a clinical application in the control of sepsis in addition to other mitochondrial diseases highlighted above.

## AOX to combat smoke-induced emphysema in the mouse

Chronic obstructive pulmonary disease (COPD) describes a number of conditions of chronic inflammatory lung disease which obstruct the airflow [214]. Most prominent conditions contributing to COPD are emphysema and chronic bronchitis. Emphysema describes a pathology in which the terminal ends of the airways (alveoli) are gradually destructed, which ultimately impairs vital gas exchange. The greatest risk factors for the development of emphysema are cigarette smoke but also other toxic gases as well as particulate matter and household air pollution [215–218]. The lack of the endogenous ability to regenerate and the almost complete absence of therapeutic measures to halt or reverse tissue damage make COPD an incurable disease whose progression can currently at best be slowed [219].

One reason for the observed cigarette smoke toxicity may be its content of detrimental oxidants [220] which cause local inflammation and consequently trigger long-term lung remodeling [221,222]. The identification of the main toxin may prove difficult since cigarette smoke is a mix of more than 5000 toxic and carcinogenic compounds [223]. Importantly, from a mitochondrial perspective, is that cigarette smoke contains carbon monoxide and hydrogen cyanide both of which are potent complex IV inhibitors. These two compounds alone potentially disrupt the electron flow through the ETC impairing ATP production, redox homeostasis, and ROS production. Indeed, previous studies have provided substantial evidence for a smoke-induced respiratory impairment [224–227]. To directly study the involvement of mitochondrial ETC dysfunction and its hierarchy in the development of COPD, we tested the role of AOX in a murine model of smoke-induced emphysema [139]. In this study, AOX attenuated the degree of lung tissue destruction and loss of lung function. To identify underlying mechanisms, embryonic fibroblasts isolated from mouse were treated with cigarette smoke condensate (CSC) [139], a well-established surrogate for cigarette smoke [228–230]. Expression of AOX increased cellular viability and decreased mitochondrial ROS level upon CSC exposure. An unexpected finding of our study was that the so-called early-phase inflammatory response, i.e. the acutely induced infiltration of macrophages and neutrophils, and the expression of cytokines upon smoke exposure was unaffected. This is surprising since a pro-inflammatory cytokine signature was considered a trigger for adverse lung remodeling [231–233]. Apparently, this assumption needs to be revisited.

## Concluding remarks and outlook

**Figure 3.**
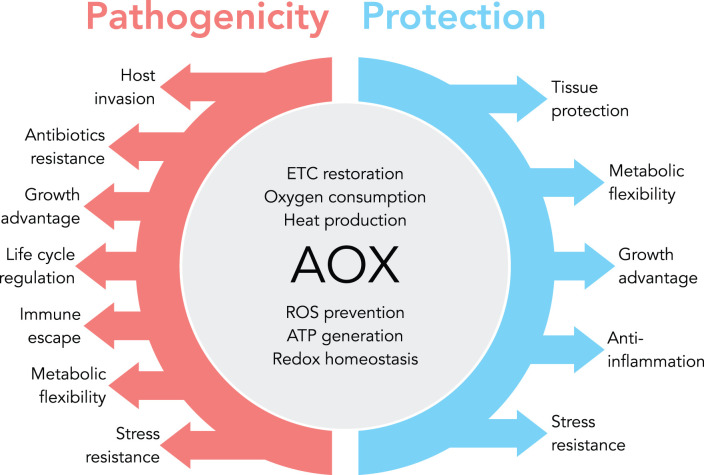
AOX is a naturally evolved stress response gene that restores electron flux through the ETC when the canonical pathway is blocked. By facilitating a bypass of the canonical ETC, it enables oxygen consumption and heat production, prevents mitochondrial ROS production, indirectly supports ATP generation by OXPHOS, and maintains cellular redox balance and TCA cycle turnover. Plants, fungi and other natural hosts use AOX to increase their metabolic flexibility and stress resistance. Pathogens utilize AOX to increase pathogenicity. Since a number of human diseases are based on ETC impairment, it has been proposed that xenotopic expression of AOX can be used as a therapeutic in human diseases to eventually alleviate disease-related side effects.

Bypassing a blocked ETC by AOX increases the viability of organisms. This mechanism helps plants, fungi and other organisms adapt to their ever-changing environment, but is also used by pathogens to evade the host's immune response (Figure 3). To protect the host while attacking a pathogen, several AOX inhibitors have been developed and are currently being tested. The fact that the AOX is naturally absent from humans can facilitate the design of targeted drugs with fewer side effects. The use of such compounds may become a ‘game changer' for many applications, whether as agrochemicals or as antibiotics to combat multidrug-resistant organisms. Interestingly, many human diseases are based on a blockade of the ETC. We and others have shown that xenotopic expression of AOX, a cross-species gene transfer, can challenge disease paradigms in appropriate disease models and, as such, AOX expression has the potential to cure lethal so-called mitochondrial diseases. However, the ultimate goal must be to optimize AOX for better applicability with tailored biochemical properties.

## Abbreviations

AD: Alzheimer's disease
AOX: alternative oxidase
APOE4: apolipoprotein E4
COPD: chronic obstructive pulmonary disease
CSC: cigarette smoke condensate
ETC: electron transport chain
PD: Parkinson's disease
SHAM: salicylhydroxamic acid
